# Production of a recombinant peroxidase in different glyco-engineered *Pichia pastoris* strains: a morphological and physiological comparison

**DOI:** 10.1186/s12934-018-1032-6

**Published:** 2018-11-24

**Authors:** Alexander Pekarsky, Lukas Veiter, Vignesh Rajamanickam, Christoph Herwig, Clemens Grünwald-Gruber, Friedrich Altmann, Oliver Spadiut

**Affiliations:** 1 Institute of Chemical, Environmental and Bioscience Engineering, Research Area Biochemical Engineering, Technische Universität Wien, Gumpendorfer Strasse 1a, 1060 Vienna, Austria; 2 Christian Doppler Laboratory for Mechanistic and Physiological Methods for Improved Bioprocesses, TU Wien, Gumpendorfer Straße 1a, 1060 Vienna, Austria; 3 Department of Chemistry, University of Natural Resources and Life Sciences, Muthgasse 18, 1190 Vienna, Austria

**Keywords:** *Pichia pastoris*, SuperMan_5_, *OCH1*, Bioreactor, Cellular agglomeration, Flow cytometry, Glycosylation, Horseradish peroxidase, Morphology

## Abstract

**Background:**

The methylotrophic yeast *Pichia pastoris* is a common host for the production of recombinant proteins. However, hypermannosylation hinders the use of recombinant proteins from yeast in most biopharmaceutical applications. Glyco-engineered yeast strains produce more homogeneously glycosylated proteins, but can be physiologically impaired and show tendencies for cellular agglomeration, hence are hard to cultivate. Further, comprehensive data regarding growth, physiology and recombinant protein production in the controlled environment of a bioreactor are scarce.

**Results:**

A Man_5_GlcNAc_2_ glycosylating and a Man_8–10_GlcNAc_2_ glycosylating strain showed similar morphological traits during methanol induced shake-flask cultivations to produce the recombinant model protein HRP C1A. Both glyco-engineered strains displayed larger single and budding cells than a wild type strain as well as strong cellular agglomeration. The cores of these agglomerates appeared to be less viable. Despite agglomeration, the Man_5_GlcNAc_2_ glycosylating strain showed superior growth, physiology and HRP C1A productivity compared to the Man_8–10_GlcNAc_2_ glycosylating strain in shake-flasks and in the bioreactor. Conducting dynamic methanol pulsing revealed that HRP C1A productivity of the Man_5_GlcNAc_2_ glycosylating strain is best at a temperature of 30 °C.

**Conclusion:**

This study provides the first comprehensive evaluation of growth, physiology and recombinant protein production of a Man_5_GlcNAc_2_ glycosylating strain in the controlled environment of a bioreactor. Furthermore, it is evident that cellular agglomeration is likely triggered by a reduced glycan length of cell surface glycans, but does not necessarily lead to lower metabolic activity and recombinant protein production. Man_5_GlcNAc_2_ glycosylated HRP C1A production is feasible, yields active protein similar to the wild type strain, but thermal stability of HRP C1A is negatively affected by reduced glycosylation.

**Electronic supplementary material:**

The online version of this article (10.1186/s12934-018-1032-6) contains supplementary material, which is available to authorized users.

## Background

The methylotrophic yeast *Pichia pastoris*, also known as *Komagataella phaffii*, is widely used for the production of recombinant proteins, due to its high productivity, the ability to grow on defined and inexpensive media and its capacity to perform post-translational modifications (e.g. [1]). Protein glycosylation is one of the most critical aspects in the recombinant production of proteins, especially of biopharmaceuticals, as it affects protein properties such as solubility, stability, biological activity, pharmacokinetics (e.g. [2]), clearance from the body and efficacy (e.g. [3]). In *P. pastoris* protein O-glycosylation still has to be fully understood, but is expected to consist of variable short, unphosphorylated/phosphorylated α-1,2- and β-1,2-mannose chains (e.g. [4]). First approaches to alter O-glycosylation in *P. pastoris* showed promising results [5]. Protein N-glycosylation, which is characterized by hypermannosylation, has been thoroughly investigated and is well understood (e.g. [6]). Initially, *N*-glycans are linked to the amido group of asparagine residues that are recognized by glycosyltransferases in the endoplasmatic reticulum (ER) at the sequence motif N-X-S/T of the proteins, where X is any amino acid but proline. After the ER, the proteins carry a Man_8_GlcNAc_2_ glycan chain, which is then subjected to hypermannosylation. The first reaction in hypermannosylation is catalyzed by an α-1,6-mannosyltransferase (Och1), which was first discovered and characterized in *S. cerevisiae* [7, 8]. Notably, its glycosylation activity of secreted and membrane proteins make it also a key enzyme for cell wall maintenance and integrity in yeast [9–12]. However, hypermannosylation hinders the use of recombinant proteins from yeasts in most biopharmaceutical applications, which is why numerous efforts focused on the humanization of the yeast glycosylation machinery [6, 13–17]. Although the humanization of yeast was accomplished over 10 years ago, only a few studies are known, in which biopharmaceutically relevant products with glyco-engineered strains were produced. Most of the strains have an *OCH1* deficiency and retain a recombinant α-1,2-mannosidase in the ER to yield mainly Man_5_GlcNAc_2_ structures [15–23].

First studies by Vervecken et al. [16] and Jacobs et al. [15] reported higher stress sensitivity of such strains leading to reduced growth, but to homogeneously (> 90%) Man_5_GlcNAc_2_ glycosylated products. In most cases, only shake-flask experiments were performed, in which comparisons to other strains or impact of product glycosylation patterns can be biased, due to the uncontrolled behavior in terms of process control (e.g. pH, dissolved oxygen) [19, 20, 22, 23]. In literature, environmental stressors are known to affect a protein’s post-translational processing [24], which highlights the importance of analyzing a protein’s properties during the controlled production in bioreactors. To our knowledge, only a few studies exist, which analyzed the behavior of Man_5_GlcNAc_2_ glycosylating *P. pastoris* strains in the controlled environment of a bioreactor [18, 21, 25]. Jacobs et al. [25] were able to produce a maximum of 760 mg L^−1^ of a murine granulocyte-macrophage colony-stimulating factor (mGM-CSF) at high cell densities upon MeOH induction of alcohol oxidase 1 promoter (P*AOX1*). Although they successfully produced almost homogeneously (> 90%) Man_5_GlcNAc_2_ glycosylated mGM-CSF, a comparison of performance to a mGM-CSF expressing wild-type strain would have been interesting. Furthermore, a decrease in productivity was observed after 40 h of MeOH induction, but was not further discussed. In another study by Smith et al. [18], the authors successfully produced recombinant human mast cell chymase (rhChymase) under the control of a glyceraldehyde-3-phosphate dehydrogenase promoter (P*GAP*). During their glycerol fed-batch cultivation, they detected chymase-like proteolytic activity after 72 h of induction, which might have resulted from physiological stress. Recently, the cotton plant proteins GbDIR2 and GhDIR3 were produced with > 90% Man_5_GlcNAc_2_ glycosylation homogeneity, but the authors observed an increased MeOH toxicity for their glyco-engineered strain compared to conventional wild-type strains [21]. Concluding, all of these research groups experienced a decrease in *Pichia’s* process performance over time. We speculate that their glyco-engineered yeast strains were physiologically impaired, due to the altered glycosylation machinery. As we have shown previously for a horseradish peroxidase C1A (HRP C1A) expressing *P. pastoris* strain, where we knocked out *OCH1* [12], an altered glycosylation machinery can have significant impacts: we found that the *OCH1* knockout strain was characterized by slow growth, increased temperature sensitivity and formation of cellular agglomerates, compared to the HRP C1A expressing wild-type strain [12]. Further analysis of this cellular agglomeration showed a decrease in cell surface glycosylation, a negatively affected budding process and indicated covalently bound cells. However, the recombinant protein was still produced and carried a much more homogeneous surface glycosylation, with the majority being Man_8_GlcNAc_2_ and Man_9_GlcNAc_2_ structures [12].

In the present study, we shed more light on the physiological impairment accompanied by glyco-engineered strains, by performing a morphological investigation and analyzing the size distribution of cellular agglomerates of differently glycosylating *P. pastoris* strains by microscopy and flow cytometry. Furthermore, we physiologically characterized a recombinant Man_5_GlcNAc_2_ glycosylating strain (SuperMan_5_) in a bioreactor during un-induced phases and MeOH induced phases, produced HRP C1A as a model product and biochemically characterized it. The used strain is based on the GlycoSwitch^®^ plasmids and is able to yield Man_5_GlcNAc_2_ glycosylated products by harboring an *OCH1* disruption and an α-1,2-mannosidase from *Trichoderma reesei* with a C-terminal HDEL signal sequence for ER retention [15]. Due to its shortened glycan pattern, a comparable morphology to the *OCH1* deficient strain was expected. We compared all results of the SuperMan_5_ strain to previously published data of a recombinant *P. pastoris* wild type (wt) (hypermannosylated product) as well as to a recombinant *P. pastoris OCH1* knockout strain (∆*OCH1*) (Man_8–10_GlcNAc_2_ glycans; [12]).

Summarizing, in this study we compared different glyco-engineered *P. pastoris* strains for morphological differences and recombinant protein production. To our knowledge, we provide the first study in literature that comprehensively describes the physiology and growth behavior of a Man_5_GlcNAc_2_ glycosylating *P. pastoris* strain in the controlled environment of a bioreactor.

## Results and discussion

### Strain characterization in shake-flask screenings

Glyco-engineered strains often show a decrease in productivity over time [18, 21, 25], which might be linked to their altered glycosylation machinery and therefore a stressed metabolism. Based on our recent findings with the ∆*OCH1* strain [12, 26], we found that cellular agglomeration and therefore an altered morphology, affected process performance over time. Therefore, we initially hypothesized that an altered glycosylation machinery might be the trigger for morphological deviations, due to an overall decrease in glycan length on the cell surface resulting in cellular agglomeration, as it was also shown for glyco-proteins [27]. Based on this hypothesis, we performed a shake-flask screening under inducing conditions to produce HRP C1A as recombinant model product in different *P. pastoris* strains and monitored cellular morphology by microscopy and flow cytometry. A hypermannosylating wt strain, the ∆*OCH1* strain (Man_8–10_GlcNAc_2_ glycans) and the SuperMan_5_ strain (Man_5_GlcNAc_2_ glycans) were compared.

We observed that the wt and SuperMan_5_ strain grew similarly in terms of OD_600_ over the whole induction time of 71 h, but observed stagnating growth for the ∆*OCH1* strain (see Additional file 1: Figure S1 for OD_600_ growth curve). At low cell densities (OD_600_ ~ 10) and after 47 h of induction, when samples for the glycosylation analysis were taken, the wt and SuperMan_5_ cultivations showed no significant difference in volumetric activity, however the ∆*OCH1* cultivation showed a significantly lower volumetric activity (Table 1). This highlighted two aspects: first, due to the lower volumetric activity and a stagnating growth, the ∆*OCH1* strain seemed to have a stressed metabolism that probably resulted in a decreased viability over time. As foam formation was more prominent for the ∆*OCH1*, partial cell lysis and higher abundance of host cell proteins in the cultivation broth was likely and was also observed in our previous studies [12, 26]. Second, the shake-flask screening data in Table 1 suggest that the performance of the SuperMan_5_ strain was not negatively affected by its altered glycosylation machinery as its performance was comparable to the wt strain.

**Table 1:** Volumetric activity of HRP C1A from wt, ∆*OCH1* and SuperMan_5_ strains from the shake-flask screening, measured in duplicates

	Volumetric activity (U mL^−1^) at OD_600_ of 10	Volumetric activity (U mL^−1^) after 47 h of induction
wt	2.20 ± 0.04	3.20 ± 0.10
∆*OCH1*	2.02 ± 0.05	2.78 ± 0.08
SuperMan_5_	2.13 ± 0.05	3.01 ± 0.09

In order to investigate strain morphology and previously observed agglomerate formation [12, 26], shake-flask samples were analysed via microscopy as well as flow cytometry.

### Microscopy

**Fig. 1:**
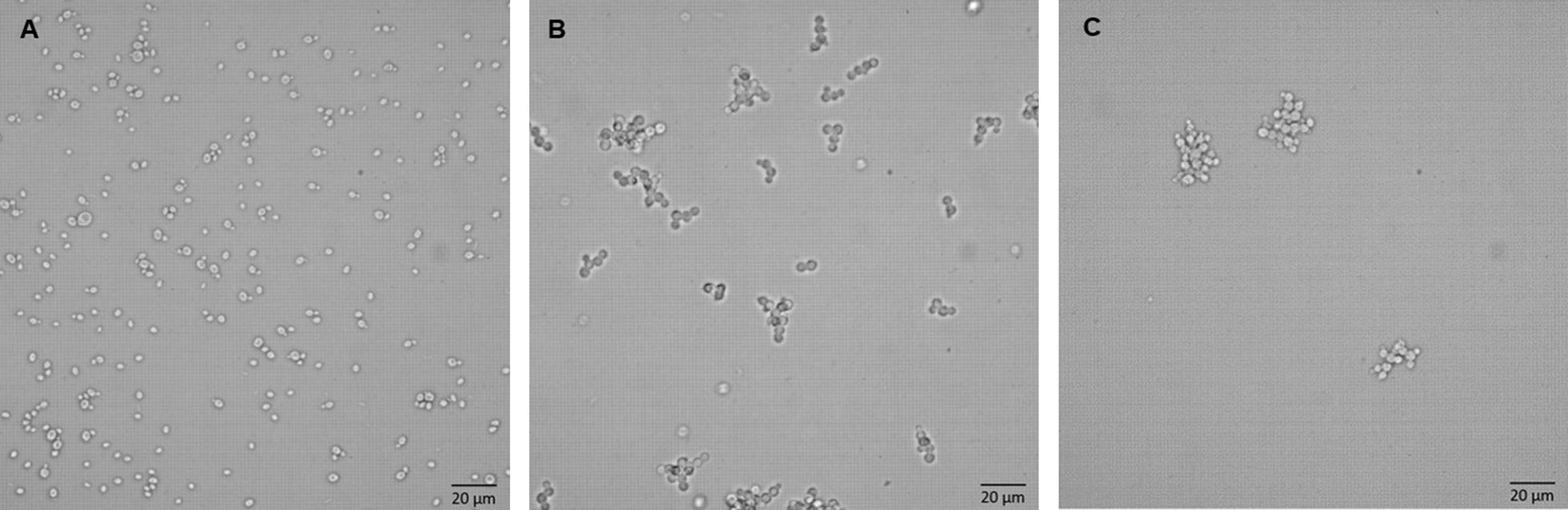
Light microscopy images taken from shake flask experiment at induction time of 11 h. Yeast cells from the different strains are shown, **A** wt, **B** ∆*OCH1*, **C** SuperMan_5_. The glyco-engineered strains in **B**, **C** show a distinct differentiation from the wt strain in **A**. Black bar signifies 20 µm

The average cell size for *P. pastoris* lies between 4 and 6 μm in average cell diameter [28]. However, conventional budding cells can also encompass two or more cells. Therefore, we defined structures smaller than 15 µm as single or budding cells and structures larger than 15 µm as agglomerates [26]. In Fig. 1, microscopic pictures from shake-flask samples during MeOH induction are displayed, which enable a clear distinction between the strains. The wt strain shows typical single and budding cells. Strong agglomerate formation was seen for both glyco-engineered strains, but the SuperMan_5_ strain seemed to have a higher degree of agglomeration. Observed agglomerates clearly exhibited multi-budded cells and spanned up to agglomerate diameters over 25 µm. Although the microscopic analysis supported our initial hypothesis that glyco-engineered strains tend to cellular agglomeration, we aimed to analyze this phenomenon with additional methods to minimize biased results. As cellular agglomeration can be triggered by sedimentation during microscopic analysis, we also used flow cytometry to analyze cell morphology under fluid conditions, which approximated the moving environment in a bioreactor or shake-flask better than conventional microscopy.

#### Flow cytometry

By using flow cytometry, not only sedimentation and possibly biased cellular agglomeration was minimized, but also a false-positive detection of loosely agglomerated cells could be minimized, due to the in-flow velocity of the cell suspension and the resulting force on the cells. Therefore, the detected cell agglomerates were thought to consist of cells, being attached to each other by strong non-covalent forces through their surface glycosylation or even by covalent bonds through an inefficient budding process [12]. These agglomerates were termed “cluster” in the evaluation of flow cytometry data.

Signal curve properties of various detector signals were used to differentiate morphological classes. As explained by Dubelaar and Gerritzen, forward scatter (FSC) and sideward scatter (SSC) signals represent size, shape and overall morphology of measured elements [29]. By using the flow cytometer, it was possible to distinguish between budding cells and cellular agglomerates. Furthermore, fluorescence signals derived from staining with propidium iodide (PI) and fluorescein diacetate (FDA) provided the means for biomass viability assessment [30]. Metabolic activity is shown by FDA treatment resulting in green fluorescence through esterase activity [31]. PI fluorescence is a result from DNA intercalation in cells with compromised membranes [28].

**Fig. 2:**
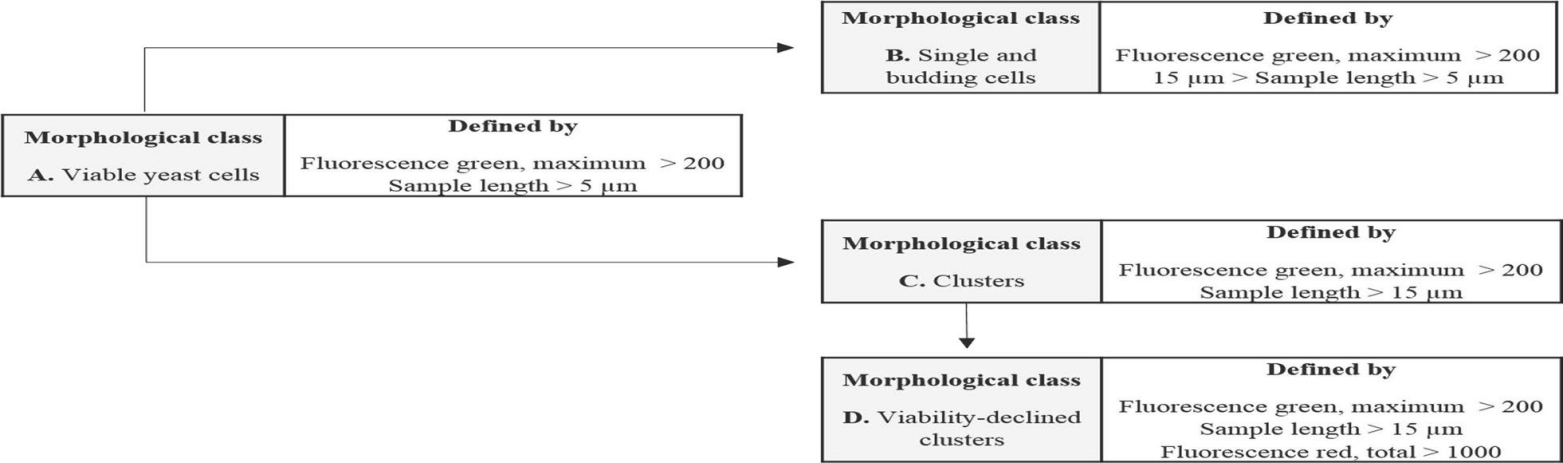
Definition of morphological classes for flow cytometry analysis

Based on initial measurements of induction medium with and without cells, a distinction of yeast cells from media background was possible and only particles above a threshold of maximum green fluorescence higher than 200, representing FDA staining, were set as viable yeast cells. In the next step, scatter plots were created and gates were set for classification. Gate setting was based on particle size in accordance with microscopic image analysis as discussed in the previous section and on our prior results with a cell agglomerate forming strain [12, 26]. The image-in-flow feature supported the visual identification of morphological classes, as single or budding cells and cluster could be distinguished. An increase in red fluorescence from PI staining indicated viability-declined agglomerates, because PI cannot cross the membrane of healthy cells (see Additional file 1: Figure S2 for comparison between viable and viability-declined clusters). Again, it was possible to set the threshold for viability-declined clusters, based on initial experiments, in which we deliberately induced cell death by heat treatment and compared the scatter plots of treated and untreated cells (data not shown). Therefore, a threshold of total red fluorescence of 1000 was set to distinguish between living and viability-declined cells. The definition of the used morphological classes for flow cytometry analysis is summarized in Fig. 2. Based on the pre-set ranges for the morphological classes, a proper distinction between different particles was possible. First, all viable yeast cells were detected, before these cells were divided into single and budding yeast cells or clusters. In a further step, these clusters were analyzed in depth, which revealed viability-declined clusters harboring a significant amount of PI permeable cells. These cells likely underwent substrate or oxygen limitation, hence the agglomerate formation led to a decrease in viability of these cells.

**Fig. 3:**
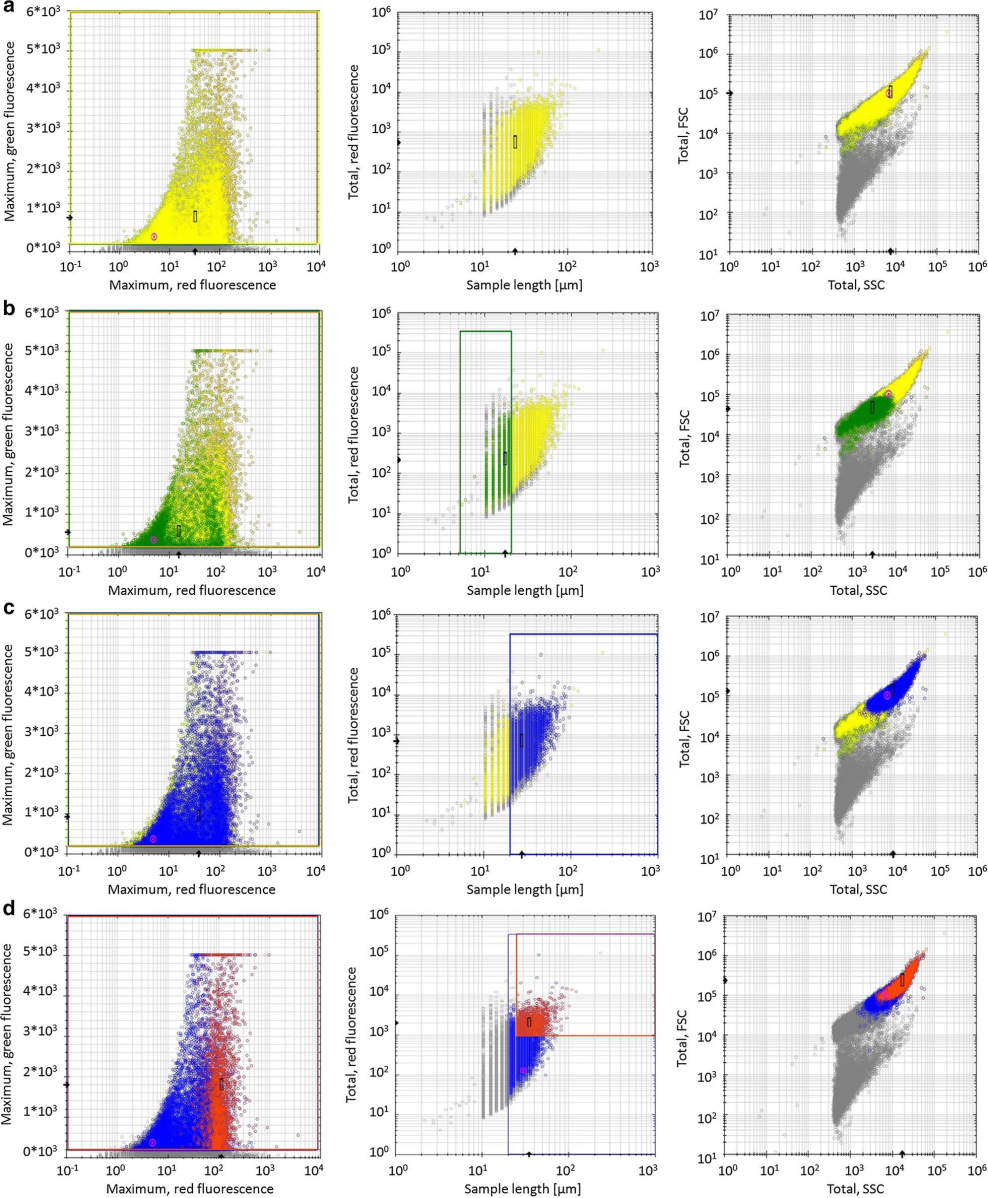
Exemplary flow cytometry scatter plots of SuperMan_5_ strain from shake-flask screening during induction at 23 h. From left to right: fluorescence green vs. fluorescence red, fluorescence red vs. sample length, FSC total vs. SSC total. From top to bottom: **a** viable yeast cells (yellow), **b** single and budding cells (green), **c** clusters (blue) and **d** viability-declined clusters (red). Rectangles signify chosen gates according to morphological classification

**Fig. 4:**
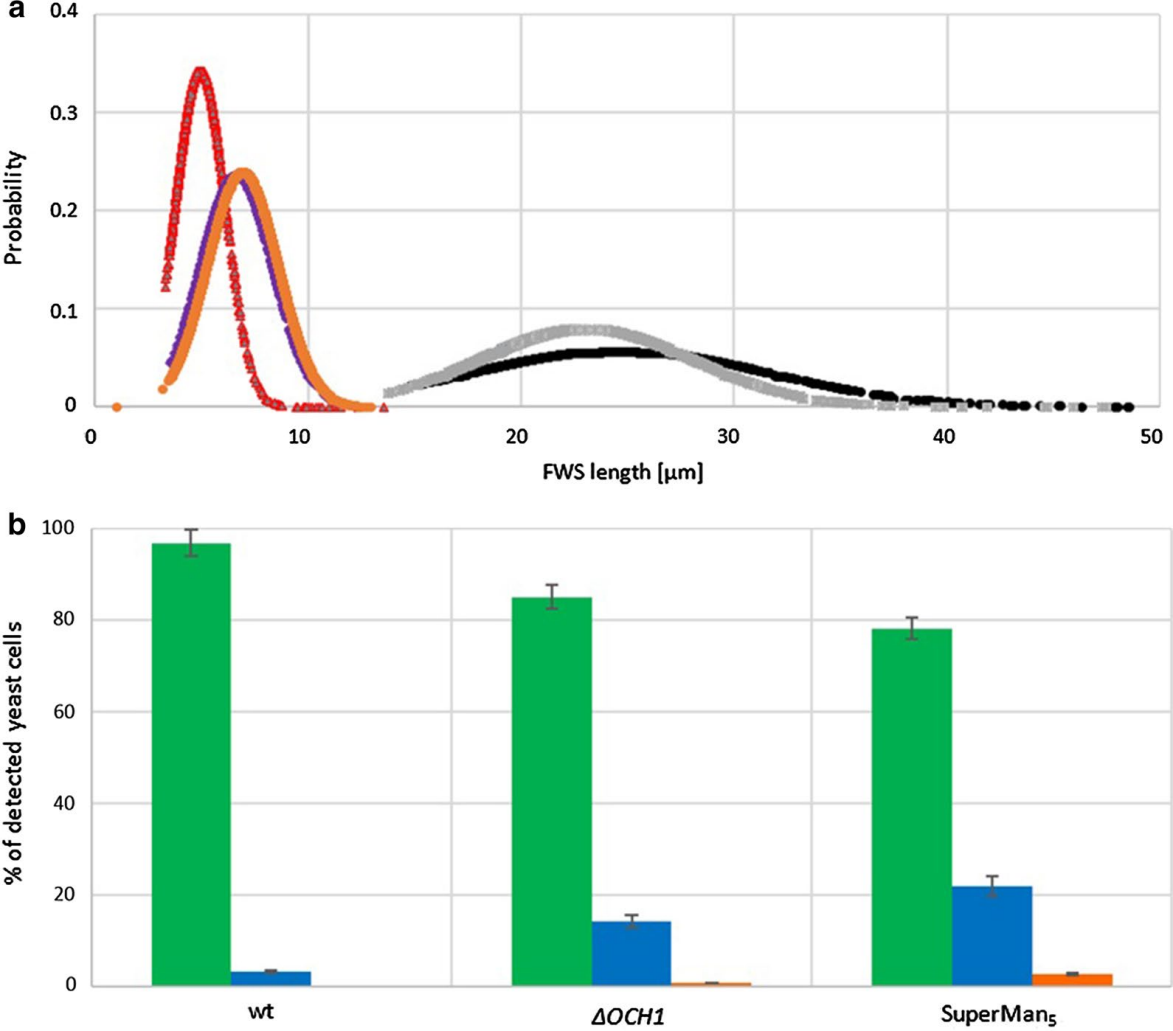
**a** Normal distribution of particle FSC length of single and budding cells and cluster for wt, *∆OCH1* and SuperMan_5_ strains at induction time of 23 h from shake-flask screening. Distribution of wt single and budding cells (red), *∆OCH1* single and budding cells (violet), SuperMan_5_ single and budding cells (orange), *∆OCH1* cluster distribution (grey), SuperMan_5_ cluster distribution (black). **b** Exemplary proportion of morphological classes in percentages of detected yeast cells at induction time of 23 h from shake-flask screening for wt, *ΔOCH1* and SuperMan_5_ strain. Bar plot shows single and budding cells (green), clusters (blue), viability-declined cluster (red). Standard deviations for **b** were derived from multiple measurements (at least 3) of single culture shake-flask samples

**Fig. 5:**
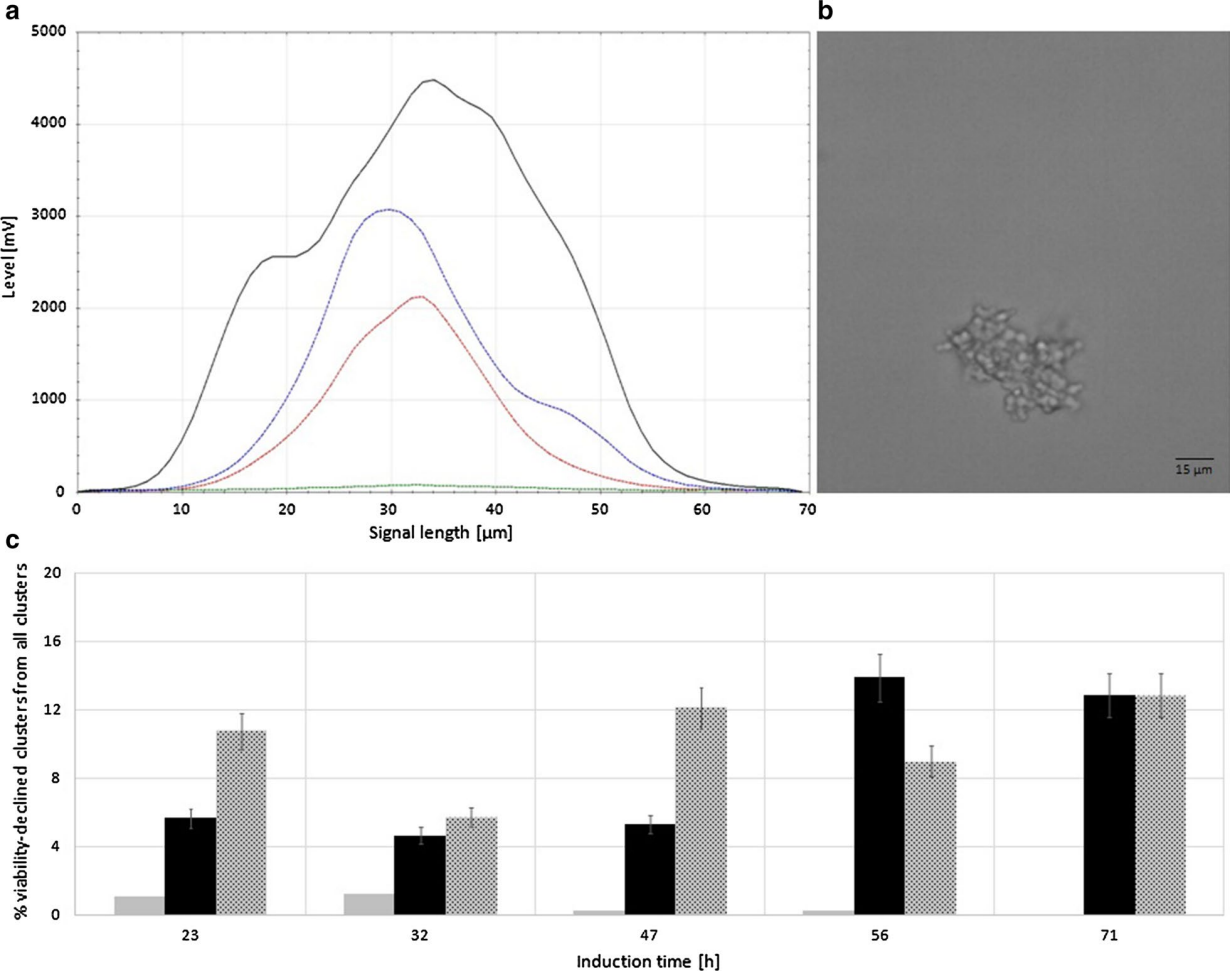
**a** Exemplary signal profile from flow cytometer of SuperMan_5_ cluster after 23 h induction time in shake-flasks. FSC (black line -), SSC (blue line –), green (green line –) and red (red line –) fluorescence signals. **b** The corresponding image-in-flow picture of the cellular cluster, black bar represents 15 µm. **c** Percentages of viability-declined clusters from all detected clusters over induction time from shake-flask screening: wt (grey bars), *∆OCH1* (black bars), SuperMan_5_ (dotted grey bars). Standard deviations for **c** were derived from multiple measurements (at least 3) of single culture shake-flask samples

The process of morphological distinction through flow cytometry is exemplarily shown for the SuperMan_5_ strain from the MeOH induced shake-flask cultivation (Fig. 3). According to the classes set in Fig. 2, viable yeast cells (Fig. 3a yellow), single and budding cells (Fig. 3b green) clusters (Fig. 3c blue) as well as viability-declined clusters (Fig. 3d red) could be distinguished. Interestingly, 23 h after induction start all three strains already showed different morphological distributions for single and budding cells and for clusters (Fig. 4a). The size distribution of wt strain for single and budding yeast was visibly narrower compared to the glyco-engineered strains. This might indicate that wt cells were less stressed. In literature, it has been reported that yeast cell size can still increase, when cellular proliferation is hindered by stress, leading to larger cells [32]. Furthermore, the size distribution of ∆*OCH1* clusters was narrower distributed and generally smaller in contrast to SuperMan_5_ clusters, hence ∆*OCH1* clusters were more tightly packed. As seen in Fig. 4b, also the wt strain was found to yield a small percentage of cluster forming cells, but with a negligible amount when compared to the glyco-engineered strains. Cluster formation of the SuperMan_5_ strain and the ∆*OCH1* strain might be triggered by the decrease in glycan length on the cell surface. It is likely that cell surface glycans help to sustain repulsive electrostatic interactions of the cells and thus prevent agglomeration, which has also been shown for glycosylated proteins in high concentrations [27]. In addition, both glyco-engineered strains showed viability-declined clusters, which correlated well to our hypothesis that cellular agglomeration can lead to limitations for inner core cells [12]. A representative signal profile of a SuperMan_5_ viability-declined cluster can be seen in Fig. 5a together with the corresponding image-in-flow of the measured cluster in Fig. 5b. The signal profile shows an increase in susceptibility to PI staining (see Additional file 1: Figure S2 for comparison between viable and viability-declined clusters), resulting in an increase of red fluorescence that corresponds to a decrease in viability of these cells. Interestingly, the SuperMan_5_ strain exhibited a stronger proportion of viability-declined clusters at the beginning of the MeOH induced shake-flask experiments (Fig. 5c). Over time, this trend diminished as the ∆*OCH1* strain increased its proportion in viability-declined clusters (Fig. 5). These results together with the fact that the mean cluster size of the glyco-engineered strains remained similar over the whole induction time (Additional file 1: Figure S3) supported the morphological similarity between these two strains. However, while the overall morphology between the ∆*OCH1* and SuperMan_5_ strain was similar, the SuperMan_5_ strain appeared to be fitter, due to its better HRP C1A production and no visible growth stagnation in the shake flasks. We hypothesized that the glyco-engineered strains differed in performance neither due to their altered glycosylation machinery nor due to their tendency to agglomerate. Both glyco-engineered strains strongly agglomerated and both were *OCH1* deficient, which suggested another reasons for the decreased performance of the ∆*OCH1* strain. Although not tested, we hypothesized that the different methods of *OCH1* inactivation could be responsible for the observed difference in performance. The ∆*OCH1* strain was generated by a knock-out procedure [12], but the SuperMan_5_ strain by an *OCH1* knock-in procedure [15, 16]. Therefore, most of the *OCH1* gene and promoter remained present, but inactive in the knock-in strain [16]. In literature, not only the Och1 protein, but also the *OCH1* gene and its promoter region are described as important factors for cell wall integrity [10] as well as oxidative- and hypo-osmotic stress tolerance [9, 11]. For example, Li and colleagues showed that the transcription factor Skn7p, which is important in stress response pathways in *S. cerevisiae*, binds upstream of the *OCH1* open reading frame and they further suggested that successful Skn7p binding promotes activation of other transcription factors. Therefore, it might be possible that binding/activation of Skn7p or other important factors were hindered in the *OCH1* knock-out strain (∆*OCH1*), which diminished its stress tolerance, but future research has to elucidate this theory. Overall, the SuperMan_5_ strain seemed to be fit for further characterization in the bioreactor and a proper production strain for Man_5_GlcNAc_2_ glycosylated HRP C1A. Additional analysis of the glycosylation pattern of HRP C1A from the different strains also indicated that the destined glycosylation was present for each of the different HRP C1A enzymes (Additional file 1: Figure S4).

Summarizing, we compared the HRP C1A production in three different *P. pastoris* strains that yielded differently glycosylated products and analysed strain morphological properties by microscopy and flow cytometry. The ∆*OCH1* and SuperMan_5_ strain showed similar morphological traits. Both glyco-engineered strains had larger single and budding cells than the wt strain and showed strong cellular agglomeration, as we had described for the ∆*OCH1* strain before [12]. This phenomenon of agglomeration is believed to be triggered by the shorter glycan structures on the cell surface and a disturbed budding process. We also found that inner core cells of these agglomerates appeared to be less viable, due to possible limitations, hence one should consider this fact when working with other glyco-engineered strains. Additionally, our results suggested that the diminished performance of the ∆*OCH1* strain did not result from the altered glycosylation machinery, but rather from different phenomena, likely including hindered stress tolerance pathways. Nevertheless, a high volumetric activity of HRP C1A was found for the SuperMan_5_ strain, which led to further strain characterization in the bioreactor.

### Physiological strain characterization in the bioreactor

Based on the prior shake-flask screening, the SuperMan_5_ strain was seen as a suitable strain for the production of Man_5_GlcNAc_2_ glycosylated HRP C1A in the controlled environment of a bioreactor. First, we characterized the SuperMan_5_ strain with our published method of conducting dynamic experiments with pulse-wise feeding during batch cultivations in the bioreactor [12, 33–36] and to our knowledge, this study represents the first comprehensive analysis of the temperature dependent physiology and growth behavior of a Man_5_GlcNAc_2_ glyco-engineered *P. pastoris* strain. The strain specific physiological parameters of SuperMan_5_ for the batch phase and the MeOH induction phase at different temperatures are summarized in Table 2.

**Table 2:** Strain specific parameters of SuperMan_5_

	SuperMan_5_
max. µ_Gly_ (h^−1^)	0.28 ± 0.03
Y_X/Gly_ (Cmol Cmol^−1^)	0.55 ± 0.03
${\text{Y}}_{{{\text{CO}}_{ 2} /{\text{Gly}}}}$ (Cmol Cmol^−1^)	0.47 ± 0.02
Δtime_adapt_ (h)	4.85

	15 °C	20 °C	25 °C	30 °C
q_MeOH_ (mmol g^−1^ h^−1^)	0.76 ± 0.12	1.17 ± 0.05	1.41 ± 0.18	1.52 ± 0.11
Y_X/MeOH_ (Cmol Cmol^−1^)	0.037 ± 0.001	0.024 ± 0.002	0.022 ± 0.006	0.033 ± 0.001
${\text{Y}}_{{{\text{CO}}_{ 2} /{\text{MeOH}}}}$ (Cmol Cmol^−1^)	0.924 ± 0.001	0.923 ± 0.048	0.961 ± 0.046	0.972 ± 0.033
C-balance	0.961 ± 0.003	0.947 ± 0.071	0.983 ± 0.074	1.005 ± 0.048
q_p_ (U g^−1^ h^−1^)	0.62 ± 0.06	0.94 ± 0.07	1.01 ± 0.06	1.05 ± 0.05

The batch phase on glycerol and the adaptation to methanol were performed at 30 °C. Subsequent methanol pulses were added at different temperatures. Parameters are defined in the footnote

*max. µ*_*Gly*_ maximum specific growth rate on glycerol, *q*_*Gly*_ specific uptake rate of glycerol during the batch, *Y*_*X/Gly*_ biomass yield on glycerol, $Y_{{CO_{2} /Gly}}$ CO_2_ yield on glycerol, *Δtime*_*adapt*_ time from first addition of methanol to a maximum in offgas activity, *q*_*MeOH*_ average specific uptake rate of methanol during consecutive methanol pulses, *q*_*P*_ specific product formation rate, *Y*_*X/MeOH*_ biomass yield on methanol, $Y_{{CO_{2} /MeOH}}$ CO_2_ yield on methanol, *C-balance* sum of biomass and CO_2_ yields

As shown in Table 2, the specific uptake rate for MeOH (q_MeOH_) increased with increasing temperature until 25 °C. The specific productivity (q_p_) showed only a significant difference at 15 °C. At 25 °C and 30 °C, the only significant difference was found in the biomass yield on MeOH. To assess the physiology of the SuperMan_5_ strain in a more comprehensive way, we compared its strain specific physiological parameters at 30 °C to our published results for a HRP C1A producing wt strain and ∆*OCH1* strain under the same conditions (Table 3).

**Table 3:** Strain specific physiological parameters of the different glyco-engineered *P. pastoris* Mut^S^ strains expressing HRP C1A as model enzyme at 30 °C

	wt [12]	∆*OCH1* [12]	SuperMan_5_
max. µ_Gly_ (h^−1^)	0.31	0.20	0.28 ± 0.03
Y_X/Gly_ (Cmol Cmol^−1^)	0.41	0.54	0.55 ± 0.03
${\text{Y}}_{{{\text{CO}}_{ 2} /{\text{Gly}}}}$ (Cmol Cmol^−1^)	0.64	0.44	0.47 ± 0.02
Δtime_adapt_ (h)	19.9	8.90	4.85
q_MeOH_ (mmol g^−1^ h^−1^)	0.70	0.43	1.52 ± 0.11
Y_X/MeOH_ (Cmol Cmol^−1^)	0.071	0.046	0.033 ± 0.001
${\text{Y}}_{{{\text{CO}}_{ 2} /{\text{MeOH}}}}$ (Cmol Cmol^−1^)	1.02	Constantly decreasing	0.97 ± 0.03
C-balance	1.09	Constantly decreasing	1.01 ± 0.05
q_p_ (U g^−1^ h^−1^)	0.77	0.50	1.05 ± 0.05

All results were gathered from a previous batch phase on glycerol, a subsequent MeOH adaptation and dynamic pulsing experiment with MeOH. Values for wt and ∆*OCH1* strain were taken from [12]

As shown in Table 3, the HRP C1A expressing *P. pastoris* strains showed differences in physiological parameters during batch on glycerol, hence their growth behaviour was already distinguishable. The SuperMan_5_ strain showed a similar maximum specific growth rate (µ_max_) to the wt strain, but the ∆OCH1 strain clearly exhibited slower growth. The above discussed shake-flask screening showed that both glyco-engineered strains have similar morphology and thus, cellular agglomeration does not seem to trigger the slow growth of the ∆*OCH1* strain, but rather hindered stress tolerance pathways. More interestingly, the glyco-engineered strains had a better conversion of substrate to biomass, as seen by a higher biomass yield and produced less CO_2_ per substrate compared to the wt strain. We hypothesized that this might be connected to the altered glycosylation machinery in both strains that led to smaller glycans. Smaller glycans also mean that less carbon is used to build sugar molecules, hence the carbon can be re-routed for biomass conversion.

All three strains behaved very differently during adaptation to MeOH and induction with MeOH. The decrease in glycan length correlated with the adaptation time (Δtime_adapt_) for MeOH, but the cause of this faster adaptation has to be elucidated yet. As also seen during the shake-flask screening, the SuperMan_5_ strain was superior during induction by yielding the best results for HRP C1A productivity and the highest q_MeOH_. Whereas ∆*OCH1* lost metabolic activity over time, shown in a constantly decreasing ${\text{Y}}_{{{\text{CO}}_{ 2} /{\text{S}}}}$ [12], the wt and SuperMan_5_ strain were easily cultivated at 30 °C for a prolonged induction time. Closing C-balances for wt and SuperMan_5_ underlined the validity of the calculated strain specific physiological data.

Summarizing, it was possible to characterize the Man_5_GlcNAc_2_ glycosylating SuperMan_5_ strain in the controlled environment of a bioreactor during un-induced batch on glycerol and induced batches on MeOH. The use of dynamic substrate pulsing made it possible to characterize the SuperMan_5_ strain at 30 °C, 25 °C, 20 °C and 15 °C during induction with MeOH in only one experiment. HRP C1A productivity was similar between 20 and 30 °C. Furthermore, a comparison between the SuperMan_5_, ∆*OCH1* and wt strain was possible at 30 °C to yield comprehensive data on the impact of glyco-engineering on growth behaviour and HRP C1A productivity. The SuperMan_5_ strain exhibited similar µ_max_ as the wt strain during batch, but the glyco-engineered strains seemed to have a more efficient substrate to biomass conversion. We hypothesized that this resulted from decreased carbon demand for the glycosylation machinery. The decrease in glycan length of protein and cell surface glycosylation of the SuperMan_5_ strain did not lead to a lower metabolic activity, growth or protein productivity compared to the wt strain.

## Enzyme characterization

### Biochemical enzyme characterization

To check whether the kinetic constants and the stability of the recombinantly produced model protein were affected by the altered glycosylation pattern, we characterized the twofold concentrated and diafiltrated HRP C1A from the SuperMan_5_ bioreactor cultivation.

We compared published results for substrate affinity and thermal stability of HRP C1A produced in the wt and ∆*OCH1* strain from our recent study (Table 4; [12]). The affinity towards the substrate ABTS was not significantly affected by altered protein glycosylation. However, the thermal stability of HRP C1A decreased clearly together with the amount of sugars attached to the protein surface. This phenomenon has already been described in literature for glycosylated proteins before [37–39].

**Table 4:** Comparison of K_M_ABTS_ and thermal stability at 60 °C between recombinant HRP C1A from wt, ∆OCH1 and SuperMan_5_

	K_M_ABTS_ (mM)	τ_1/2_ (s)
wt [12]	2.40	384
∆*OCH1* [12]	2.03	198
SuperMan_5_	2.04 ± 0.34	121

Values for wt and ∆*OCH1* produced HRP C1A were taken from [12]

Summarizing, we analyzed enzyme kinetics and thermal stability of HRP C1A from the SuperMan_5_ strain. Although enzyme affinity towards ABTS was comparable to wt and ∆*OCH1* products, the thermal stability was diminished 3-fold compared to the HRP C1A from the wt strain, hence proofing that protein stability is strongly affected by glycosylation.

## Conclusion

In this study, we report the first comprehensive evaluation of growth, physiology and recombinant protein productivity of a Man_5_GlcNAc_2_ glycosylating *P. pastoris* strain (SuperMan_5_) in the controlled environment of a bioreactor. The strain showed superior growth, physiology and HRP C1A productivity compared to a Man_8–10_GlcNAc_2_ glycosylating ∆*OCH1* strain.

Additionally, we shed more light on the often seen impairment in process performance of glyco-engineered strains in a detailed morphological study: Flow cytometry and microscopic analysis revealed the formation of cellular agglomerates with impaired viability of inner core cells. Although agglomeration was prominent in both glyco-engineered strains, our results suggest that a decreased process performance might not necessarily derive from the altered glycosylation machinery. It can be rather attributed to an additional metabolic burden, like hindered stress tolerance pathways, which can make strains more sensitive to environmental stressors.

## Methods

### Chemicals

Enzymes, deoxynucleotide triphosphates and Phusion™ high-fidelity DNA-polymerase was obtained from ThermoFisher Scientific (Vienna, Austria). 2,2′-azino-bis(3-ethylbenzthiazoline-6-sulfonic acid) diammonium salt (ABTS) and hemin were purchased from Sigma-Aldrich (Vienna, Austria). Difco™ yeast nitrogen base w/o amino acids (YNB), Difco™ yeast nitrogen base w/o amino acids and ammonia sulfate (YNB2), Bacto™ tryptone and Bacto™ yeast extract were purchased from Becton–Dickinson (Vienna, Austria). Zeocin™ was purchased from InvivoGen (Toulouse, France) via Eubio (Vienna, Austria).

### Microorganisms

For this study, a *HRP C1A* gene, codon-optimized for *P. pastoris*, was ordered from GenScript (Nanjing, China) and cloned into a pPICZαC vector, providing a Zeocin™ (Zeo) resistance gene as well as an α-prepro mating signal sequence from *Saccharomyces cerevisiae* for product secretion, using standard methods. Correct integration was verified by sequencing. The pPICZαC vector was successfully integrated in a *P. pastoris* GS115 strain (*HIS*^+^, *pep4Δ*, *aox1∆*), kindly provided by Biogrammatics, Inc. (California, United States) and should yield mainly Man_5_GlcNAc_2_ glycosylated HRP C1A after transformation (SuperMan_5_) [15]. The Och1 deficiency of the SuperMan_5_ strain is based on the disruption, but not deletion of the *OCH1* gene. The strain CBS 7435 (identical to NRRL Y-11430 or ATCC 76273) was used as a benchmark wild type strain (wt), which yields natively hypermannosylated HRP C1A [12]. As described in our previous study, we used a genetically engineered wt strain harboring a knock-out deletion of the *OCH1* gene (∆*OCH1*) to avoid hypermannosylation that yielded mainly Man_8–10_GlcNAc_2_ glycosylated HRP C1A upon transformation [12]. Both, the wt and ∆*OCH1* strain contained a pPpT4_S, which contained the codon-optimized *HRP C1A* gene under equal conditions [12, 40]. Therefore, all resulting strains had a Mut^S^ phenotype, expressed and secreted HRP C1A upon induction of the *AOX1* promoter with MeOH.

### Culture media

The growth medium [buffered medium with glycerol for yeast (BMGY)] for the shake-flask screenings contained: 10 g L^−1^ yeast extract, 20 g L^−1^ peptone, 13.4 g L^−1^ YNB2, 4 mg L^−1^ d(+)-biotin, 10 g L^−1^ glycerol and 100 mL of a 1 M potassium phosphate buffer pH 6.0. The induction medium [buffered medium with MeOH for yeast (BMMY)] for the shake-flask screenings contained: 10 g L^−1^ yeast extract, 20 g L^−1^ peptone, 13.4 g L^−1^ YNB2, 4 mg L^−1^ d(+)-biotin, 5 g L^−1^ MeOH and 100 mL of a 1 M potassium phosphate buffer pH 6.0. The preculture medium for the bioreactor cultivations [yeast nitrogen base medium (YNBM)] contained: 20 g L^−1^ α-d(+)-glucose monohydrate, 3.4 g L^−1^ YNB2, 10 g L^−1^ (NH_4_)_2_SO_4_, 0.4 g L^−1^ d(+)-biotin, 0.1 M potassium phosphate buffer, pH 6.0. Trace element solution (PTM1) for the bioreactor cultivation contained: 6 g L^−1^ CuSO_4_·5H_2_O, 0.08 g L^−1^ NaCI, 3 g L^−1^ MnSO_4_·H_2_O, 0.2 g L^−1^ Na_2_MoO_4_·2H_2_O, 0.02 g L^−1^ H_3_BO_3_, 0.5 g L^−1^ CoCl_2_, 20 g L^−1^ ZnCl_2_, 65 g L^−1^ FeSO_4_·7H_2_O, 0.2 g L^−1^ d(+)-biotin, 5 mL L^−1^ 95–98% H_2_SO_4_. Basal salt medium (BSM) for the bioreactor cultivations contained: 60 g L^−1^ glycerol, 1.17 g L^−1^ CaSO_4_·2H_2_O, 18.2 g L^−1^ K_2_SO_4_, 14.9 g L^−1^ MgSO_4_·7H_2_O, 4.13 g L^−1^ KOH, 26.7 mL L^−1^ 85% (v/v) o-phosphoric acid, 0.2 mL L^−1^ Antifoam Struktol J650, 4.35 mL L^−1^ PTM1, NH_4_OH as N-source. pH was maintained by using 12.5% NH_3_,_aq_. Throughout all shake-flask cultivations, Zeocin™ was used in a concentration of 50 µg mL^−1^.

### Strain selection

After transformation, 10 Zeo resistant clones were picked and grown overnight in 10 mL BMGY-Zeo medium in 100 mL baffled shake-flasks at 230 rpm and 30 °C. Then, cells were harvested by centrifugation (1800×*g*, 4 °C, 10 min) and re-suspended in BMMY-Zeo for adaptation of the cells to MeOH. Again, cells were grown at 230 rpm and 30 °C. Recombinant protein production was induced by adding 1.5% (v/v) pulses of pure MeOH supplemented with 12 mL PTM1/L MeOH to the culture each day, for 5 days. Each day, a sample was taken and analyzed for OD_600_, total protein content in the cell-free cultivation broth (Bradford assay) as well as the presence of recombinant HRP C1A by SDS-PAGE. A recombinant *Pichia pastoris* strain carrying the empty pPICZαC vector was included as negative control in all experiments.

### Analysis of strain morphology and glycosylation

To understand a possible impact of the genotype and phenotype on overall strain physiology and productivity, an initial shake-flask screening, including morphological analysis was performed. The strain morphology was analyzed under inducing conditions for the wt, ∆*OCH1* and SuperMan_5_ strain. In parallel, growth and product formation were monitored to assure product presence for later glycosylation pattern analysis.

#### Shake-flask screening

A fresh cryo tube (− 80 °C) was thawed for each HRP C1A containing strain, added to 200 mL BMGY-Zeo medium in a 1000 mL shake-flask and incubated at 28 °C and 230 rpm overnight. The next day, 50 mL of each culture were transferred to 450 mL BMMY-Zeo, including also 10 µM Hemin (Heme) to ease HRP C1A induction [41]. Induced cultures were grown in 2.5 L baffled flasks and a working volume of 500 mL. For comparability, HRP C1A induction was performed at 28 °C for all 3 strains. To assure complete depletion of the initial C-source (glycerol) and accurate adaptation to the inducing C-source in the shake-flasks (MeOH), cells were grown for 23 h in BMMY-Zeo-Heme before the first MeOH pulse was given. MeOH pulses were given each day as 1% (v/v) with PTM1 (12 mL L^−1^ MeOH). Sampling of the cultures was done approximately every 12 h. After 47 h of induction, 100 mL of each culture were harvested, centrifuged (4000×*g*, 10 min, 4 °C), the cell free supernatant was concentrated 20× with a 10 kDa centrifugal filter membrane (Amicon^®^Ultra—15) tube (Merck Millipore Ltd., Carrigtwohill, IRL) and stored at − 20 °C for further analysis. Enzyme activity and total protein content of the concentrates were measured and aliquots of the concentrates were used for identification of the respective HRP C1A glycosylation pattern of each strain. However, the total induction time of the shake-flask cultures was 71 h to further monitor growth and the morphological behavior of the different strains.

#### Microscopy

Twenty microlitres of the cultivation broth were pipetted onto a standard glass slide (25 × 75 mm) and then covered with an extra-large cover slide (24 × 60 mm). Images were recorded at a 40× magnification with a five megapixel microscopy CCD colour camera (Olympus, Austria). These images were used as a rough estimation of cell agglomerate formation and agglomerate diameter.

#### Flow cytometry

Samples of the shake-flask screening were diluted in phosphate buffered saline (PBS) (2.65 g L^−1^ CaCl_2_ solution, 0.2 g L^−1^ KCl, 0.2 g L^−1^ KH_2_PO_4_, 0.1 g L^−1^ MgCl·6 H_2_O, 8 g L^−1^ NaCl and 0.764 g L^−1^ Na_2_HPO_4_·2H_2_O at pH 6.5) to an OD_600_ of 1. Then, 0.5 µL of 20 mM propidium iodide stock in dimethyl sulfoxide (both from Sigma Aldrich, St. Louis, United States) and 5 µL of 12 mM fluorescein diacetate (Sigma Aldrich, St. Louis, United States) stock in acetone were added to 0.5 mL of the cell suspension. After 10 min incubation in the dark at room temperature, the sample was further diluted (1:10 in PBS) for flow cytometric analysis.

A CytoSense flow cytometer (CytoBuoy, Woerden, Netherlands) with two forward scatter (FSC), one sideward scatter (SSC) and two fluorescence channels (green, red) was used for single cell analysis. The implemented laser had a wavelength of 488 nm. The configuration of the emission wavelength filter set was 515–562 ± 5 nm for the green fluorescence channel (used for fluorescein diacetate) and 605–720 ± 5 nm for the red fluorescence channel (used for propidium iodide). The flow cytometer was equipped with a PixeLINK PL-B741 1.3 MP monochrome camera for image-in-flow acquisition, which made real-time imaging of cell agglomerates possible. For data evaluation, the software CytoClus3 (CytoBuoy, Woerden, Netherlands) was used.

The CytoSense flow cytometer provides multiple spatially resolved data points per channel per particle. This signal is achieved for both scatter channels as well as for green and red fluorescence channels [29], which is the basis for multiple curve parameters. Except for length parameters in µm, all parameters are in arbitrary units, as the user can set the sensitivity of the detector. The following parameters were used for the distinction of morphological classes: maximum (maximum of signal curve), total (area under curve), length (length of the signal) and sample length (length of signal above trigger level). Furthermore, image-in-flow feature enabled visual identification of yeast agglomerates, termed clusters. It should be noted that while FSC signals are closely linked to particle size (sample length), FSC length signals do not always correspond entirely to sample length due to overlays of other signals, which was seen during calibration with defined beads.

### Glycosylation analysis

A glycopeptide analysis using an LC–ESI–MS system was performed, as we have reported before [12, 42]. The concentrated samples of the shake-flask screening were digested in solution. The proteins were S-alkylated with iodoacetamide and digested with trypsin (Promega, Madison, United States). The peptide mixtures were loaded on a BioBasic C18 column (BioBasic-18, 150 × 0.32 mm, 5 µm; ThermoFisher Scientific, Vienna, Austria) using 80 mM ammonium formiate buffer as the aqueous solvent. A gradient from 5% B (B: 80% acetonitrile) to 40% B in 45 min was applied, followed by a 15 min gradient from 40% B to 90% B that facilitates elution of large peptides, at a flow rate of 6 µL min^−1^. Detection was performed with a QTOF MS (Bruker maXis 4G) equipped with the standard ESI source in positive ion, DDA mode (= switching to MSMS mode for eluting peaks). MS-scans were recorded (range 150–2200 Da) and the 3 highest peaks were selected for fragmentation. Instrument calibration was performed using an ESI calibration mixture (Agilent, Santa Clara, United States). The nine possible glycopeptides were identified as sets of peaks consisting of the peptide moiety and the attached N-glycan varying in the number of HexNAc, hexose and phosphate residues. The theoretical masses of these glycopeptides were determined with a spreadsheet using the monoisotopic masses for amino acids and monosaccharides. Manual glycopeptide searches were made using DataAnalysis 4.0 (Bruker, Billerica, United States).

### Bioreactor cultivations

After conducting the shake-flask screening, we characterized the recombinant SuperMan_5_ strain in terms of physiology, biomass growth and productivity using a dynamic strategy of conducting MeOH pulses during batch cultivations in the controlled environment of a bioreactor, which we have described several times before [12, 33–36]. This cultivation was used for subsequent purification to perform product kinetics and thermal stability analysis.

#### Preculture

Frozen stocks (− 80 °C) from working cell banks were incubated in 100 mL of YNBM-Zeo in 1 L shake-flasks at 30 °C and 230 rpm for 24 h. The preculture was transferred aseptically to the respective culture vessel. The inoculation volume was 10% of the final starting volume.

#### Cultivation

Batch cultivation was carried out in a 5 L working volume Labfors glass bioreactor (Infors, Bottmingen, Switzerland). BSM was sterilized in the bioreactor and pH was adjusted to pH 5.0 by using 12.5% NH_3,aq_ after autoclavation. Sterile filtered PTM1 was transferred to the reactor aseptically. pH and dissolved oxygen probes were calibrated prior to cultivation start. Dissolved oxygen (dO_2_) was measured with a sterilizable polarographic dissolved oxygen electrode (Mettler Toledo, Vienna, Austria) and maintained above 20% throughout the cultivation. The pH was measured with a sterilizable electrode (Mettler Toledo, Vienna, Austria) and maintained constant at pH 5.0 with a step controller using 12.5% NH_3,aq_. Base consumption was determined gravimetrically. Agitation was fixed to 1495 rpm. The culture was aerated with 2.0 vvm dried air and offgas of the culture was measured by using an infrared cell for CO_2_ and a paramagnetic cell for O_2_ concentration (Servomax, Egg, Switzerland). Temperature, pH, dO_2_, agitation as well as CO_2_ and O_2_ in the offgas were measured on-line and logged in a process information management system (PIMS Lucullus; Applikon Biotechnology, Delft, Netherlands).

The end of the initial batch phase at 30 °C and therefore complete glycerol consumption was indicated by an increase in dO_2_, a drop in offgas CO_2_ and an increase in offgas O_2_. The first MeOH pulse (adaptation pulse) of a final concentration of 0.5% (v/v) was conducted with MeOH supplemented with 12 mL PTM1 per 1 L of added MeOH (MeOH/PTM1 pulse). Subsequently, at least two MeOH/PTM1 pulses were given to 1% (v/v) at 30 °C, then 25 °C, 20 °C and finally 15 °C. For each pulse, at least two samples were taken to determine the concentrations of MeOH and product as well as dry cell weight (DCW) and OD_600_ to calculate the strain specific physiological parameters. Induction was carried out in the presence of 1 mM hemin, which was added prior to the adaptation pulse [43].

### Sample analysis

#### Analysis of growth- and expression-parameters

Dry cell weight (DCW) was determined by centrifugation of 5 mL culture broth (4000×*g*, 4 °C, 10 min), washing the pellet twice with 5 mL water and subsequent drying for 72 h at 105 °C. Determination was performed in triplicates. OD_600_ of the culture broth was measured in duplicates using a spectrophotometer (Genesys 20; ThermoFisher Scientific, Vienna, Austria). The activity of HRP C1A in the cell free supernatant was determined with a CuBiAn XC enzymatic robot (Optocell, Bielefeld, Germany) in duplicates. Cell free samples (60 µL) were added to 840 µL of 1 mM ABTS in 50 mM potassium phosphate buffer, pH 6.5. The reaction mixture was incubated for 5 min at 37 °C and was started by the addition of 100 µL of 0.078% H_2_O_2_. Changes of absorbance at 420 nm were measured for 180 s and rates were calculated. Calibration was done by using commercially available horseradish peroxidase (Type VI-A, P6782; Sigma-Aldrich, Vienna, Austria,) as standard at six different concentrations (0.02; 0.05; 0.1; 0.25; 0.5 and 1.0 U mL^−1^). Protein concentration of cell free supernatant was determined at 595 nm using the Bradford Protein Assay Kit (Bio-Rad Laboratories GmbH, Vienna, Austria) with bovine serum albumin (protein standard; micro standard, liquid; P0914; Sigma Aldrich, Vienna, Austria) as standard.

#### Substrate concentrations

Concentration of glycerol and MeOH was determined in cell free samples of the bioreactor cultivation by HPLC (Agilent Technologies, Santa Clara, United States) equipped with a Supelco guard column, a Supelco gel C-610H ion-exchange column (Sigma-Aldrich, Vienna, Austria) and a refractive index detector (Agilent Technologies, Santa Clara, United States). The mobile phase was 0.1% H_3_PO_4_ with a constant flow rate of 0.5 mL min^−1^ and the system was run isocratically. Calibration was done by measuring standard points in the range of 0.1–10 g L^−1^ glycerol and MeOH.

#### Data analysis

Strain characteristic parameters of the bioreactor cultivation were determined at a carbon dioxide evolution rate (CER) above 2.5 mmol g^−1^ h^−1^ during each MeOH pulse. Along the observed standard deviation for the single measurement, the error was propagated to the specific rates (q_s_ and q_p)_ as well as to the yield coefficients. The error of determination of the specific rates and the yields was therefore set to 10% and 5%, respectively for single measurement-derived values like seen in the batch phase [34]. For the pulse experiments, the average value and the standard deviation were used as two pulses were given for each temperature.

### Enzyme characterization

#### Kinetic constants

A cell free bioreactor supernatant with HRP C1A from the SuperMan_5_ strain was twofold concentrated and diafiltrated with buffer (500 mM NaCl, 20 mM NaOAc, pH 6.0) [44, 45]. Protein concentration of the HRP C1A preparation was determined at 595 nm using the Bradford Protein Assay Kit (Bio-Rad Laboratories GmbH, Austria) with bovine serum albumin as standard. The kinetic constants for ABTS and H_2_O_2_ were determined. The reaction was started by adding 10 μL enzyme solution (1.0 mg mL^−1^) to 990 μL reaction buffer containing either ABTS in varying concentrations (0.01–5 mM) and 1 mM H_2_O_2_ or H_2_O_2_ in varying concentrations (0.001–0.5 mM) and 5 mM ABTS in 50 mM potassium phosphate buffer at pH 6.5. The change in absorbance at 420 nm was recorded in a spectrophotometer UV-1601 (Shimadzu, Japan) at 30 °C. Absorption curves were recorded with a software program (UVPC Optional Kinetics; Shimadzu, Japan). Measurements were performed in triplicates.

#### Thermal stability

The purified enzyme solution was incubated at 60 °C. At different time points, aliquots were withdrawn, the solutions were immediately cooled and centrifuged (20,000×*g*, 15 min) to pellet precipitated proteins and the remaining catalytic activity in the supernatants was measured [46].

## Additional file

**Additional file 1: Figure S1.** OD_600_ over induction time in shake-flask experiment, black-filled circle = wt, unfilled white circle = SuperMan_5_, grey triangle = ∆*OCH1*. **Figure S2.** Comparison of signal profiles from flow cytometer. FSC (black line -), SSC (blue line --), green (green line --) and red (red line --) fluorescence signals of SuperMan_5_ clusters after 23 h induction time in shake-flasks. In viability-declined cluster (A) a clear increase in red fluorescence is visible from PI staining in contrast to the viable cluster (B). **Figure S3.** Mean cluster size of *∆OCH1* (black bars) and SuperMan_5_ (dotted grey bars) over induction time in shake-flask cultivation. Standard deviations are derived from multiple measurements (at least 3) of single culture shake-flask samples. **Figure S4.** ESI–MS spectra of HRP glycopeptides at n-glycosylation site 7. The triply (or doubly) charged site 7 N-glycopeptide GLIQSDQELFSSP**NAT**DTIPLVR of natural HRP and HRP produced in a ∆OCH1, in SuperMan_5_ and in wt *P. pastoris* are shown. The wt_HRP exhibits also phosphorylated versions (P) of the oligomannosidic glycans.

## Abbreviations

FSC: flow cytometry forward scatter signal
SSC: flow cytometry sideward scatter signal
max. µ_Gly_: maximum specific growth rate on glycerol (h^−1^)
Y_X/Gly_: biomass yield on glycerol (Cmol Cmol^−1^)
${\text{Y}}_{{{\text{CO}}_{ 2} /{\text{Gly}}}}$: CO_2_ yield on glycerol (Cmol Cmol^−1^)
Δtime_adapt_: time from first MeOH addition to maximum offgas activity (h)
q_MeOH_: average specific uptake rate of MeOH during MeOH pulses (mmol g^−1^ h^−1^)
q_P_: specific product formation rate (U g^−1^ h^−1^)
Y_X/MeOH_: biomass yield on MeOH (Cmol Cmol^−1^)
${\text{Y}}_{{{\text{CO}}_{ 2} /{\text{MeOH}}}}$: CO_2_ yield on MeOH (Cmol Cmol^−1^)
C-balance: sum of biomass and CO_2_ yields
