# Supplementary material for: Production of a recombinant peroxidase in different glyco-engineered Pichia pastoris strains: a morphological and physiological comparison
Source: Microb Cell Fact. 2018 Nov 24;17:183. doi: 10.1186/s12934-018-1032-6 (PMC6260843; doi:10.1186/s12934-018-1032-6)
Supplement: Supplementary file 1 — Additional file 1: Figure S1. OD600 over induction time in shake-flask experiment, black-filled circle = wt, unfilled white circle = SuperMan5, grey triangle = ∆OCH1. Figure S2. Comparison of signal profiles from flow cytometer. FSC (black line -), SSC (blue line --), green (green line --) and red (red line --) fluorescence signals of SuperMan5 clusters after 23 h induction time in shake-flasks. In viability-declined cluster (A) a clear increase in red fluorescence is visible from PI staining in contrast to the viable cluster (B). Figure S3. Mean cluster size of ∆OCH1 (black bars) and SuperMan5 (dotted grey bars) over induction time in shake-flask cultivation. Standard deviations are derived from multiple measurements (at least 3) of single culture shake-flask samples. Figure S4. ESI–MS spectra of HRP glycopeptides at n-glycosylation site 7. The triply (or doubly) charged site 7 N-glycopeptide GLIQSDQELFSSPNATDTIPLVR of natural HRP and HRP produced in a ∆OCH1, in SuperMan5 and in wt P. pastoris are shown. The wt_HRP exhibits also phosphorylated versions (P) of the oligomannosidic glycans. [file 12934_2018_1032_MOESM1_ESM.docx]

**Additional file for**

**Production of a recombinant peroxidase in different glyco-engineered *Pichia pastoris* strains – a morphological and physiological comparison**

Alexander Pekarsky^1,+^, Lukas Veiter^1,2,+^, Vignesh Rajamanickam^1,2^, Christoph Herwig^1,2^, Clemens Grünwald-Gruber^3^, Friedrich Altmann^3^ and Oliver Spadiut^1*^

^1^ Technische Universität Wien, Institute of Chemical, Environmental and Bioscience Engineering, Research Area Biochemical Engineering, Gumpendorfer Strasse 1a, 1060 Vienna, Austria

^2^ Christian Doppler Laboratory for Mechanistic and Physiological Methods for Improved Bioprocesses, TU Wien, Gumpendorfer Straße 1a, 1060 Vienna, Austria

^3^ Department of Chemistry, University of Natural Resources and Life Sciences, Muthgasse 18, 1190 Vienna, Austria

+ Equal contribution

^*^ Correspondence: Oliver Spadiut, TU Wien, Institute of Chemical, Environmental and Bioscience Engineering, Research Area Biochemical Engineering, Gumpendorfer Strasse 1a, 1060 Vienna, Austria. Tel: +43 1 58801 166473, Fax: +43 1 58801 166980, Email: oliver.spadiut@tuwien.ac.at


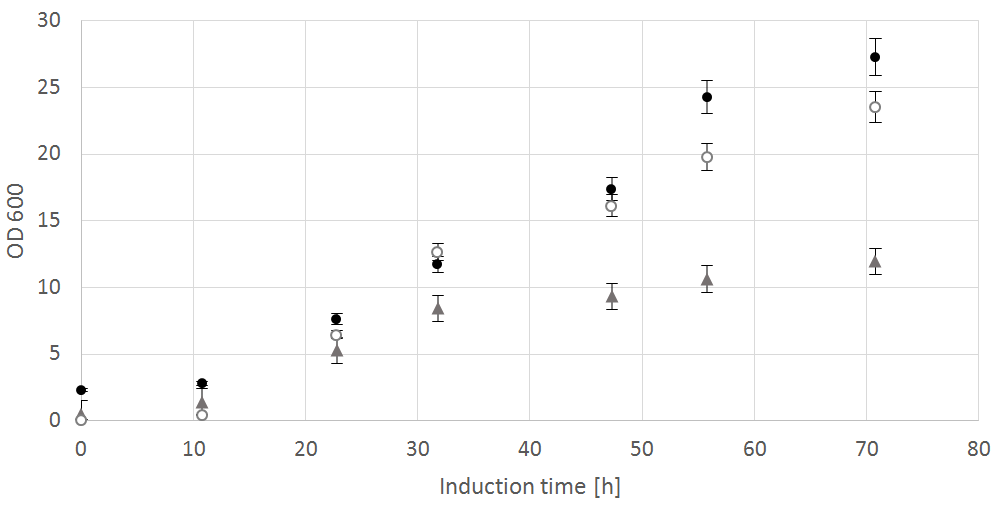


**Figure S1:** OD_600_ over induction time in shake-flask experiment, black-filled circle = wt, unfilled white circle = SuperMan_5_, grey triangle = ∆*OCH1*.


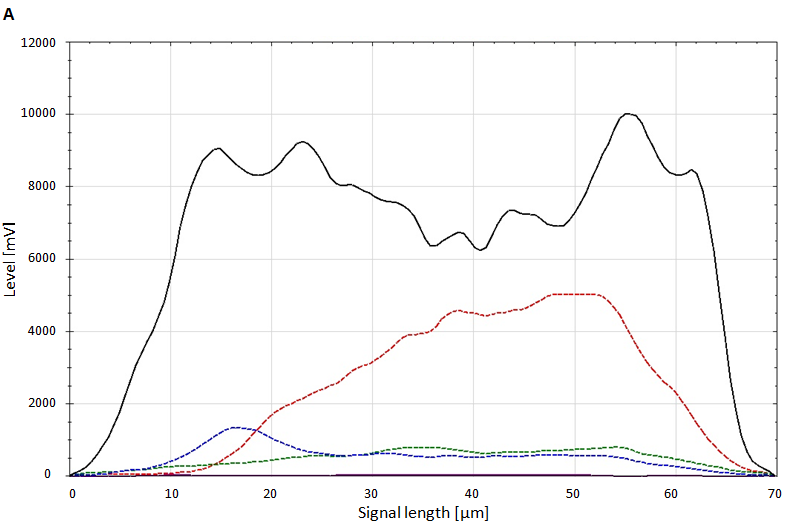

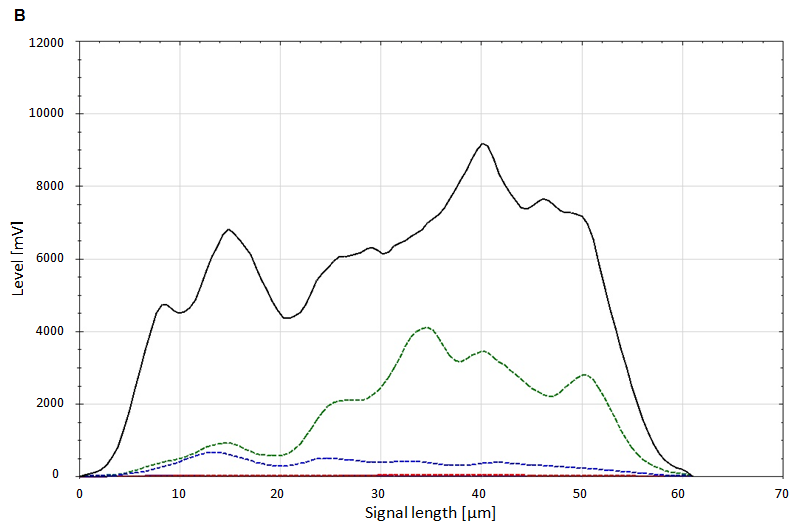


**Figure S2:** Comparison of signal profiles from flow cytometer. FSC (black line -), SSC (blue line --), green (green line --) and red (red line --) fluorescence signals of SuperMan_5_ clusters after 23 h induction time in shake-flasks. In viability-declined cluster (A) a clear increase in red fluorescence is visible from PI staining in contrast to the viable cluster (B).


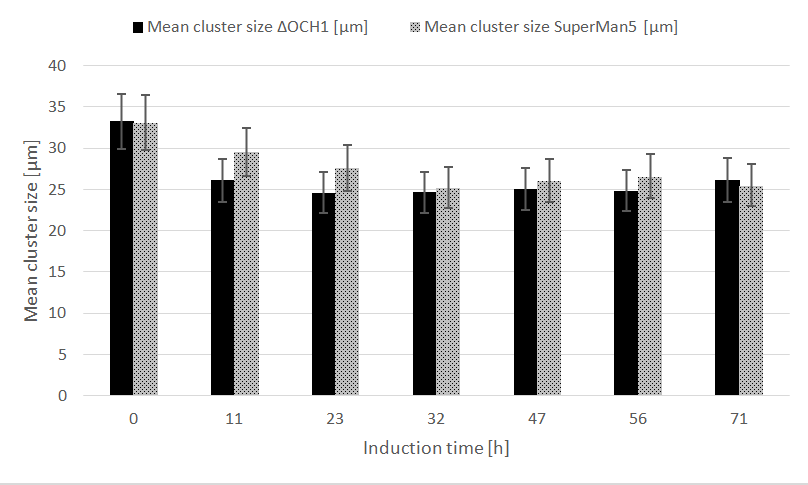


**Figure S3**: Mean cluster size of *∆OCH1* (black bars) and SuperMan_5_ (dotted grey bars) over induction time in shake-flask cultivation. Standard deviations are derived from multiple measurements (at least 3) of single culture shake-flask samples.


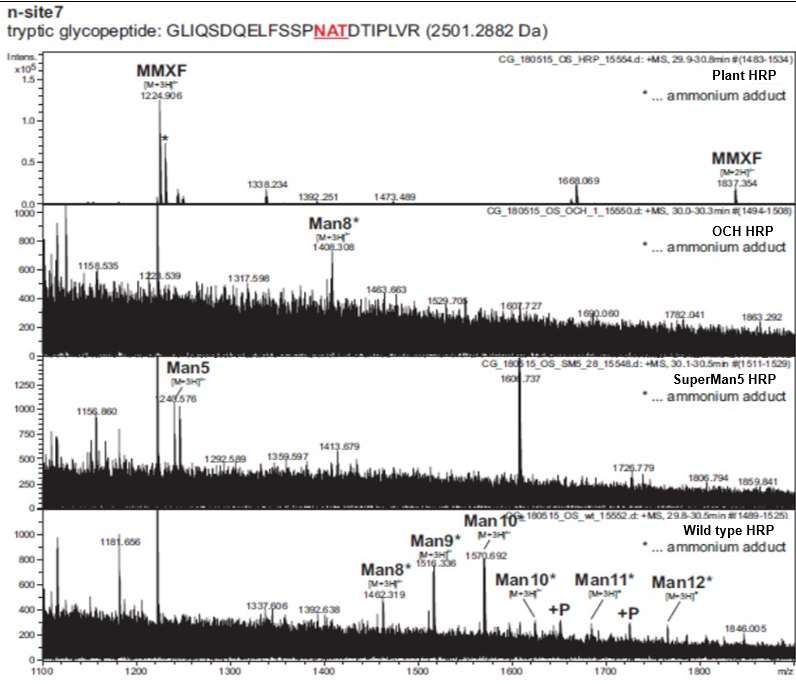


**Figure S4:** ESI-MS spectra of HRP glycopeptides at n-glycosylation site 7. The triply (or doubly) charged site 7 N-glycopeptide GLIQSDQELFSSP**NAT**DTIPLVR of natural HRP and HRP produced in a ∆OCH1, in SuperMan_5_ and in wt *P. pastoris* are shown. The wt_HRP exhibits also phosphorylated versions (P) of the oligomannosidic glycans.

To avoid interference by glycoproteins other than HRP, the glycosylation status of the HRP variants was deduced from glycopeptides, as shown for glycosite 7 (GLIQSDQELFSSPNATDTIPLVR). While natural HRP carries the plant typical MMXF structure with xylose and fucose and only 3 mannose residues, HRP C1A produced by wt
*P. pastoris* exhibited a very heterogeneous pattern in which Man_9_ and Man_10_ glycans predominated and some phosphorylated glycans were also found. HRP C1A from the ∆OCH1 strain contained mainly the Man_8_ structure and HRP C1A from the SuperMan_5_ strain was modified by Man_5_ as expected.
